# Liposomes as carriers for garlic oil delivery to increase anti-inflammatory and antioxidant activities in mice with ALI

**DOI:** 10.3389/ebm.2026.10800

**Published:** 2026-01-21

**Authors:** Ruilin Hou, Xiaowei Zhang, Jiaming Zhang, Wenping Zhang

**Affiliations:** 1 Drug/Medical Device Clinical Trial Institution Office, General Hospital of Ningxia Medical University, Yinchuan, Ningxia, China; 2 College of Pharmacy, Ningxia Medical University, Yinchuan, Ningxia, China

**Keywords:** acute lung injury, garlic oil, inflammatory cytokines, liposomes, oxidative stress

## Abstract

ALI/ARDS are clinical syndromes with diverse etiological origins and are characterized by high mortality rates and a lack of specific therapeutic options. Garlic oil (GO) has been utilized in both culinary and medicinal applications for millennia. However, its complex chemical composition and inherent instability have limited further development and clinical application. We aimed to encapsulate GO within liposomes to increase its solubility and stability. The therapeutic efficacy of GO-loaded liposomes (GO-lips) against LPS-induced ALI was subsequently evaluated *in vivo*. A novel GO-lip formulation was developed, and its preparation process was optimized to ensure its stability and bioavailability. A murine model of LPS-induced ALI was established. The animals were randomly assigned to the normal control, LPS model, GO treatment, or GO-lip treatment group. Therapeutic outcomes were evaluated by lung tissue histopathology, inflammatory cytokine quantification and oxidative stress biomarker measurement. PCR and molecular dynamics simulations were used to verify the ALI treatment-related pathways influenced by GO-lips. We successfully developed GO-lips using a novel fabrication method. GO-lips demonstrated favorable physicochemical characteristics, with a mean particle diameter of 175 ± 3 nm, a PDI of 0.27 ± 0.02, and an encapsulation efficiency of 70.74 ± 2.11%. Compared with the LPS model group, the GO-lip treatment group exhibited significant protection against LPS-induced ALI. GO-lips demonstrated greater efficacy than free GO, as evidenced by the improved lung histopathology, reduced pulmonary edema, decreased inflammatory responses, and attenuated oxidative stress. PCR analysis demonstrated that GO-lips significantly protect mice primarily via Nrf2 pathway activation. These findings suggest that liposomal encapsulation of GO increases its anti-inflammatory and antioxidant activities, protecting against LPS-induced ALI. This research offers a novel clinical therapeutic approach for ALI and contributes to foundational knowledge supporting the development and utilization of GO-derived formulations.

## Impact statement

Acute Lung Injury (ALI) and its more severe manifestation, Acute Respiratory Distress Syndrome (ARDS), represent prevalent and critical respiratory conditions characterized by high mortality rates. These disorders impose significant economic and psychological burdens on both patients and society. Currently, no effective treatment exists for ALI, and conventional glucocorticoid therapies are often accompanied by severe adverse effects. In the present study, garlic oil was administered using liposome-based nanodelivery technology, which serves to mitigate adverse drug reactions while simultaneously improving the therapeutic efficacy in the management of ALI.

## Introduction

Acute lung injury (ALI) is a rapidly progressive and severe pulmonary disorder primarily caused by bacterial and viral infections. Clinically, ALI is characterized by an exaggerated inflammatory response both within the lungs and systemically, resulting in disruption of the pulmonary endothelial barrier, impaired alveolar fluid clearance, and widespread lung tissue damage. The acute nature of ALI often results in severe dyspnea within a short timeframe, and can rapidly progress to acute respiratory distress syndrome (ARDS) within hours [1, 2]. ALI has a high incidence rate, a considerable risk of complications, and substantial treatment costs, making it one of the most prevalent and critical conditions encountered in intensive care units [3]. The pathophysiology of ALI involves complex interplay among several interrelated mechanisms, such as dysregulated inflammatory responses, oxidative stress imbalance, epithelial‒endothelial barrier disruption, and aberrant cellular apoptosis [4]. Collectively, these processes compromise the structural integrity of the alveolar‒capillary barrier and impair pulmonary gas exchange. The intricacy of the interactions among inflammation, oxidative stress, and apoptosis highlights the complexity of ALI pathogenesis. Sustained inflammatory and oxidative challenges promote apoptosis in alveolar epithelial and endothelial cells, thereby hindering tissue repair and exacerbating barrier dysfunction. Notably, the NF-κB-mediated inflammatory signaling pathway and the Nrf2/ARE-mediated antioxidant pathway have been identified as principal molecular regulators of the progression of ALI [5]. These pathways operate in conjunction with intricate crosstalk among multiple signaling cascades. Consequently, biomarkers such as tumor necrosis factor-alpha (TNF-α), various interleukins, and malondialdehyde (MDA) are frequently employed for comprehensive, multidimensional assessment of ALI. This disease imposes considerable psychological and physiological burdens on patients and their families and poses a significant global health threat. Consequently, the development of novel, safe, and effective pharmacological therapies for ALI/ARDS is urgently needed.

**FIGURE 1:**
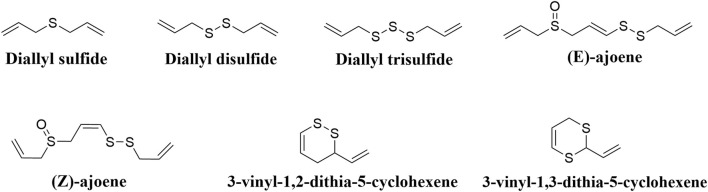
Sulfur-containing organic compounds present in GO.

*Allium sativum* L., commonly known as garlic, is an annual bulbous herb belonging to the Liliaceae family and originating from Central and South Asia. Historically, garlic has been utilized for millennia as a functional food, culinary spice, flavoring agent, and traditional medicinal plant. Despite its longstanding use in ethnomedicine, systematic investigations into its bioactive constituents have gained momentum only in recent decades [6]. The primary bioactive compounds in garlic are organosulfur compounds, which are also responsible for its characteristic pungent odor [7]. The key organosulfur constituents include diallyl sulfide (DAS), diallyl disulfide (DADS), and diallyl trisulfide (DATS), all of which demonstrate a wide range of pharmacological properties as shown in Figure 1 [8, 9]. Recent studies have extensively documented various physiological effects of garlic oil (GO), such as anti-atherosclerotic, antihypertensive, antibacterial, anticancer, and immunomodulatory effects, which are attributed predominantly to antioxidant and anti-inflammatory mechanisms [10]. However, the clinical application of GO is substantially limited by its complex chemical composition, poor solubility, instability, and pronounced pungency. Consequently, contemporary studies have concentrated on increasing the stability and bioavailability of garlic oil, attenuating its irritant properties, and optimizing its therapeutic and nutritional potential in clinical applications.

Liposomes (Lips) are nanoscale spherical vesicles that are composed primarily of phospholipids, cholesterol, and various auxiliary components; they are characterized by their nonimmunogenicity, low likelihood of causing allergic reactions, and high safety [11]. The PEG layer in GO loaded liposomes increases circulation time and provides a stealth sheath that stabilizes the drug delivery system in blood and in storage [12]. A good example of this approach is Doxil (a liposomal drug formulation that is used to deliver chemotherapeutic doxorubicin into the cancer cells). Doxil was approved by the FDA in 1995 [13]. As a mature drug delivery system, liposomes are capable of encapsulating a diverse array of compounds ranging from small molecules to large biological macromolecules, and they have been shown to significantly increase drug delivery efficiency, therapeutic efficacy and effectively reducing the side effects of drugs across a broad spectrum of disease [14]. While, by exploiting the enhanced permeability and retention (EPR) effect, which is enabled by the increased pulmonary vascular permeability in ALI, Lips with particle sizes ranging from 100 to 200 nm can accumulate at sites of lung injury via vascular gaps. This accumulation facilitates an increase in the local concentration of the therapeutic agent [15]. Consequently, liposomes were chosen as the delivery system to generate experimental data supporting the commercialization of GO nanoformulations. In subsequent studies, we intend to develop GO nanomicelles and nanoemulsions.

In this study, for the first time, liposomes composed of DSPC, cholesterol, and DSPE-MPEG2000 were used to deliver GO to ameliorate lipopolysaccharide (LPS)-induced acute lung injury. Here, GO-loaded liposomes (GO-lips) were prepared, and their morphology, size, encapsulation efficiency, and stability were systematically characterized. Moreover, we conducted an in-depth investigation into the ability of GO-lips to increase the protective efficacy of GO against LPS-induced ALI. The findings revealed that the protective mechanism of GO-lips is facilitated through the activation of Nrf2, which subsequently promotes the upregulation of antioxidant gene expression. This cascade effectively mitigates oxidative stress-induced tissue damage and suppresses the ROS-mediated inflammatory cytokine storm in pulmonary tissues. This preliminary study provides further evidence that PEG-stabilized liposomes may serve as promising carriers for hydrophobic liquid drugs.

## Materials and methods

### Chemical reagents

The following compounds were used in this study: GO (Yuanye Co. Ltd., Shanghai, China; CAS: 8008-99-9), DSPC (CAS: 816-94-4), DSPE-MPEG2000 (CAS: 147867-65-0), cholesterol (CAS: 57-88-5), all procured from A.V.T. Co. Ltd. (Shanghai, China), chloroform (Chronchem Co. Ltd., Sichuan, China; CAS: 67-66-3), methanol (Thermo Fisher Scientific, USA; CAS: 67-56-1), formic acid (Macklin, Shanghai, China; CAS: 64-18-6), HEPES (Amresco, CAS: 7365-45-9), potassium phosphate monobasic (Guangnuo Co. Ltd., Shanghai, China; CAS: 7778-77-0), potassium phosphate dibasic (Damao Co. Ltd., Tianjin, China; CAS: 7758-11-4), and dimethyl sulfoxide (Biotopped, Beijing, China; CAS: 67-68-5).

### Preparation and characterization of garlic oil-loaded PEG-stabilized liposomes

#### Preparation of garlic oil-loaded PEG-stabilized liposomes

Garlic oil-loaded PEG-stabilized liposomes (GO-lips) were prepared via the thin-film dispersion method with a DSPC: cholesterol: MPEG_2K_-DSPE molar ratio of 0.151:0.099:0.014. Briefly, 114.98 mg of DSPC, 38.3 mg of DSPE-MPEG2000, and 38.29 mg of cholesterol were accurately weighed and transferred into a 50 mL round-bottom flask containing a dry magnetic stirrer. The mixture was thoroughly dissolved in chloroform, after which an aliquot of 10 μL of GO was added. During gentle stirring for 2 h, a homogeneous and transparent lipid mixture was formed, and precautions were taken to avoid splashing the mixture onto the flask walls. The organic solvent was subsequently removed by slow evaporation using a rotary evaporator under reduced pressure at 40 °C. During solvent volatilization, the phospholipid molecules assembled into a thin and compact drug-loaded lipid film on the surface of the flask. The experimental temperature was optimized on the basis of the physicochemical properties of phospholipids to ensure complete dissolution while minimizing the risk of thermal degradation, as excessive temperatures may promote phospholipid oxidation and hydrolysis, thereby compromising the quality of the resulting film. The product was sealed to maintain its dryness and stored at 4 °C for a period of more than 3 days.

#### Characterization of GO-loaded liposomes

The solution was dissolved in selected solvents and subjected to low-temperature ultrasonication to obtain liposomes. The mean particle size of the liposomes was determined using a Malvern Zetasizer Nano ZS90 (Malvern Nano ZS, Malvern, UK). Briefly, 1 mL of the GO-loaded liposome mixture was transferred into a dish and was subsequently analyzed using a Marvin laser particle size analyzer to determine the particle size distribution.

The morphology of the liposomes was characterized via transmission electron microscopy (TEM). Briefly, a single drop of the liposome sample was carefully deposited onto a copper grid coated with a carbon membrane. The samples were allowed to air dry at ambient temperature for 30 min to ensure complete evaporation of the solvent. The samples were subsequently imaged using a Hitachi H-600 transmission electron microscope operated at an accelerating voltage of 200 kV. Micrographs were acquired at a magnification of ×20,000 with an accelerating voltage of 200 kV.

GO-lip were dissolved in deionized water, HEPES buffer, or phosphate buffer (pH 5.8), and stability assessments were conducted at controlled temperatures (4 °C, room temperature, and 40 °C) to validate the encapsulation efficiency and stability. The GO concentration was quantitatively determined using high-performance liquid chromatography (HPLC) after mixing with methanol at a 1:1 ratio. Following identification of the optimal solvent system, the micelles were reconstituted in deionized water and separated by centrifugation at 14,000 rpm for 10 min, which resulted in a precipitated phase containing encapsulated GO. The precipitate was subsequently resolubilized in DMSO through a sequential process involving 5 min of vortex mixing and 30 min of sonication, with this dissolution cycle repeated three times to ensure complete dissociation of the liposomes. Both the supernatant and the DMSO-dissolved precipitate fractions were mixed with an equal volume of methanol prior to HPLC analysis for precise determination of the encapsulation efficiency.

### Animal experiments

All animal procedures were performed in strict accordance with the national standards for the care and use of laboratory animals as outlined in the Guidelines for Welfare and Ethical Review of Laboratory Animals (GB/T35892-2018, China). The study protocol was approved by the Animal Care and Use Committee of the General Hospital of Ningxia Medical University (approval number: KYLL-2025-0079). To establish the LPS-induced ALI model, 6- to 8-week-old male ICR mice were obtained from the Animal Experiment Center of Ningxia Medical University. The animals were housed under controlled conditions, which included a 12-h light/dark cycle, an ambient temperature of 22 ± 2 °C, and a relative humidity of 50% ± 10%. Prior to experimentation, the mice were provided *ad libitum* access to standard dry chow and tap water and were acclimated for 1 week. The mice were subsequently randomly assigned to one of four groups (n = 10 for each group): the control, LPS, LPS plus GO-liposome (50 mg/kg, administered 4 h post-LPS administration), and LPS plus GO (50 mg/kg, administered 4 h post-LPS administration) groups. LPS (O111:B4; Sigma‒Aldrich, USA) was dissolved in precooled PBS (pH 7.4). 4 h after the intraperitoneal administration of 15 mg/kg LPS, the GO-loaded liposome formulation or GO was administered via tail vein injection at a dose of 50 mg/kg [16]. 2 h following the intravenous administration of GO, all mice were anesthetized using pentobarbital at a dosage of 60 mg/kg and subsequently euthanized humanely via CO_2_ inhalation. In accordance with international ethical guidelines aimed at minimizing animal distress, lung tissues, blood samples, and bronchoalveolar lavage fluid (BALF) were systematically collected.

### Evaluation of pulmonary edema via the wet/dry weight ratio and lung coefficient

After 6 h of modeling, the mice were euthanized, the left lung was harvested via thoracotomy, blotted dry with filter paper, and placed in a preweighed EP tube, after which the wet weight was recorded and labeled. The sample was subsequently dried in an oven at 80 °C for 48 h until complete dehydration was achieved, after which the dry weight was recorded. The wet/dry weight ratio (W/D) of the lung tissue and the lung coefficient were calculated, and these ratios were used to determine the lung water content, i.e., the degree of pulmonary edema.

### Histological analysis of pulmonary tissue

After 6 h of modeling, the mice were euthanized for tissue collection. For mice not subjected to bronchoalveolar lavage, the fur was initially moistened with alcohol swabs and carefully removed to expose the thoracic cavity. Subsequently, a small portion of the left lung lobe was excised with meticulous attention to preserve structural integrity in order to avoid artifacts that could interfere with data interpretation. The harvested tissue was immediately fixed with 10% formalin for 24–48 h. Following fixation, the samples were subjected to dehydration using a graded series of ethanol solutions with increasing concentrations. The tissue was subsequently embedded in paraffin, and after solidification, serial sections of 5 μm thickness were prepared. Hematoxylin and eosin (H&E) staining was subsequently performed to facilitate microscopic examination of pathological alterations within the lung tissue. The pathological scores for lung injury were assessed according to previously established criteria [16].

### Measurement of the total protein content, inflammatory cell count and cytokine levels in BALF

Following euthanasia, the thoracic cavity was surgically accessed, and the left bronchus was ligated. Subsequently, 0.6 mL of ice-cold PBS was delivered into the right lung lobe via a sterile blunt needle to collect BALF. Each mouse was injected with a total volume of 1.5 mL of ice-cold PBS. The total protein concentration in the collected BALF was quantified using a bicinchoninic acid (BCA) protein assay kit (SW201-02; Beijing Qihai Biotechnology Co., Ltd.) in accordance with the manufacturer’s protocol, and this value served as an indicator of pulmonary permeability. Inflammatory cells were stained using a Wright-Giemsa staining kit for 20 min at room temperature (D010-1-2; Nanjing Jiancheng Bioengineering Institute) and visualized by light microscopy.

### Enzyme-linked immunosorbent assay (ELISA)

Bronchoalveolar lavage fluid and lung tissue homogenates were centrifuged at 12,000 rpm for 10 min to separate the supernatant. The concentrations of TNF-α, IL-4, IL-6, and IL-10 in both the BALF, serum, lung tissue samples were determined using ELISA kits (catalog numbers 88-7324, 88-7044, 88-7064, and 88-7105; Thermo Fisher Scientific, USA). All procedures were conducted in strict accordance with the manufacturers’ protocols. Absorbance readings were obtained at 450 nm using a microplate reader, and cytokine concentrations were determined on the basis of the corresponding standard curves.

### Measurement of NO release

The *in vivo* concentration of nitric oxide was determined utilizing a nitric oxide detection kit (S0021; Beyotime Biotechnology Co., Ltd., Shanghai, China). All procedures were conducted in strict accordance with the manufacturer’s protocol. Absorbance measurements were obtained at 540 nm using a microplate reader, and nitric oxide concentrations were quantified on the basis of a standard calibration curve.

### Assessment of oxidative stress in the ALI model

After 6 h of modeling, the mice were euthanized, and the right lung lobe was excised. The tissue was immediately immersed in ice-cold PBS, promptly homogenized, and centrifuged at 12,000 rpm for 10 min to obtain the supernatant for subsequent oxidative stress assays. The total antioxidant capacity (T-AOC; kit S0121), MDA concentration (kit S0131S), superoxide dismutase (SOD) activity (kit S0101S), and hydrogen peroxide concentration (H_2_O_2_; kit S0051) in both lung tissue homogenates and serum were quantified with kits purchased from Beyotime (Shanghai, China) following the manufacturer’s protocols. Samples were aliquoted into 96-well plates, and absorbance readings were obtained using a microplate reader. The concentrations of each analyte were calculated in accordance with the instructions of the corresponding kit. All the procedures adhered strictly to the manufacturer’s recommended protocols.

### Real-time reverse transcription–PCR (qRT‒PCR) analysis of mRNA expression

In this study, total RNA was first isolated from lung tissue samples utilizing AXYGEN buffer (Wujiang, China). cDNA synthesis was subsequently carried out using an All-in-One First-Strand cDNA Synthesis SuperMix kit (TrsanBiotechgen, China). qRT‒PCR analysis was performed using a Tip Green qPCR SuperMix Kit (TrsanBiotechgen, China) with the primer sequences listed in Table 1. GAPDH served as the internal control for normalization purposes, and relative mRNA expression levels were calculated using the 2^−ΔΔCT^ method.

**TABLE 1:** Primer sequences used for quantitative real-time PCR.

Gene	Forward	Reverse
Nrf2	TCT​CCT​CGC​TGG​AAA​AAG​AA	AAT​GTG​CTG​GCT​GTG​CTT​TA
HO-1	CAA​GCC​GAG​AAT​GCT​GAG​TTC​ATG	GCA​AGG​GAT​GAT​TTC​CTG​CCA​G
NQO1	TCAGCCAATCAGCGTTC	CTC​CTT​CAT​GGC​GTA​GTT​G
GPX4	CAG​GCA​AGA​CCG​AAG​TAA​ACT​AC	CCG​AAC​TGG​TTA​CAC​GGG​AA
SOD	ATTGACGTGTGGGAGCA	AATGTGGCCGTGAGTGA
TNF-α	CAG​GTT​CTC​TTC​AAG​GGA​CAA​GGC	TGA​CGG​CAG​AGA​GGA​GGT​TGA​C
IL-1β	TGA​AGT​TGA​CGG​ACC​CCA​AAA​GAT	GTT​GAT​GTG​CTG​CTG​CGA​GAT​TTG
IL-6	AGA​CTT​CCA​TCC​AGT​TGC​CTT​CTT​G	TCT​GTT​GGG​AGT​GGT​ATC​CTC​TGT​C
iNOS	AGA​CCC​AGG​AGT​GTT​CAC​AGA​CC	CAT​TGG​CCA​GCT​GCT​TTT​GC
GAPDH	GGT​TGT​CTC​CTG​CGA​CTT​CA	TGG​TCC​AGG​GTT​TCT​TAC​TCC

### Molecular dynamics simulations

GROMACS software was employed for molecular dynamics (MD) simulations. A simulation system was developed by combining the target protein with diallyl trisulfide (DATS), the principal small molecule component of garlic oil. The simulations were carried out under isothermal–isobaric (NPT) ensemble conditions with the application of periodic boundary conditions. The assembled complex was subjected to a 50000 ps molecular dynamics run, during which conformational snapshots were recorded at 10-picosecond intervals. Key parameters, including the root mean square deviation (RMSD), root mean square fluctuation (RMSF), and radius of gyration (Rg), were computed to assess the structural stability and flexibility of the complex. Furthermore, Gibbs free energy landscape analysis was employed to identify the lowest-energy conformational state, thereby confirming the presence and stability of the interaction between the Nrf2 protein and the natural compound DATS [17].

### Statistical analysis

One-way ANOVA was used for statistical analysis. All the data are presented as the means ± SEMs, and GraphPad Prism version 10.0 (GraphPad Software, Inc., San Diego, CA, USA) was used for data analysis. After ANOVA, *post hoc* multiple comparisons were performed with Tukey’s honestly significant difference test to determine the significance of the differences among the subgroups unless otherwise indicated. *p* < 0.05 was considered to indicate a significant difference, and n.s. was used to indicate a nonsignificant difference.

## Results

### Preparation and Characterization of GO-Loaded PEG-Stabilized liposomes

**FIGURE 2:**
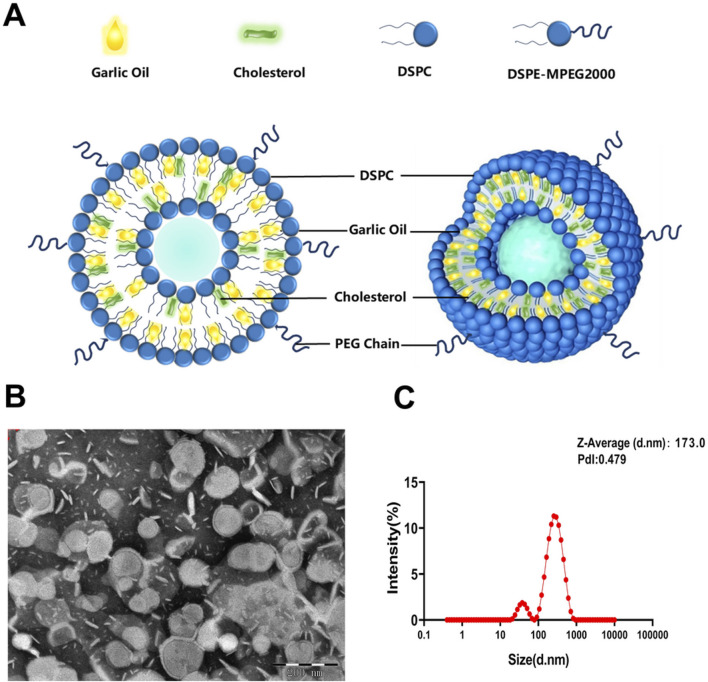
Preparation and Characterization of GO-Loaded PEG-Stabilized Liposomes. Spatial and planar structures of GO -loaded PEG-stabilized lipsomes (GO-lip). **(A)** Lipids with dark blue head groups symbolize phospholipids, those with blue-black head groups represent PEG-lipids, the yellow parts represent GO, and the green parts symbolize cholesterol. **(B)** TEM images of the GO loaded lipsomes. The scale bar on the TEM image is 200 nm. **(C)** Dynamic light scattering analysis of GO-lip. The results demonstrated that we successfully constructed GO-lip.

GO-loaded liposomes (GO-lips) were prepared using the thin-film dispersion method combined with ultrasonic hydration. GO was incorporated into the hydrophobic lipid bilayer. A schematic representation of the GO-lip nanostructure is shown in Figure 2A. The liposome exhibits a spherical configuration composed of DSPC and cholesterol, with a hydrophilic polyethylene glycol (PEG) edge chain formed by the DSPE-MPEG2000 incorporated into its structure, which stabilizes the hydrophobic periphery. The TEM images (Figure 2B) and particle size distribution analysis results (Figure 2C) confirmed the successful fabrication of GO-lips with a predominantly spherical morphology. The average particle diameter was measured to be 175 ± 3 nm, with a polydispersity index (PDI) of a PDI of 0.27 ± 0.02, a zeta potential of −0.292 ± 0.007 mV. The encapsulation efficiency of GO within the liposomes was approximately 70.74 ± 2.11%. Furthermore, owing to the increased density of GO compared with the buffer solution, the formulation exhibited increased stability, and the concentration of GO-lips was effectively increased through centrifugation.

### GO-lips effectively ameliorated LPS-induced ALI

**FIGURE 3:**
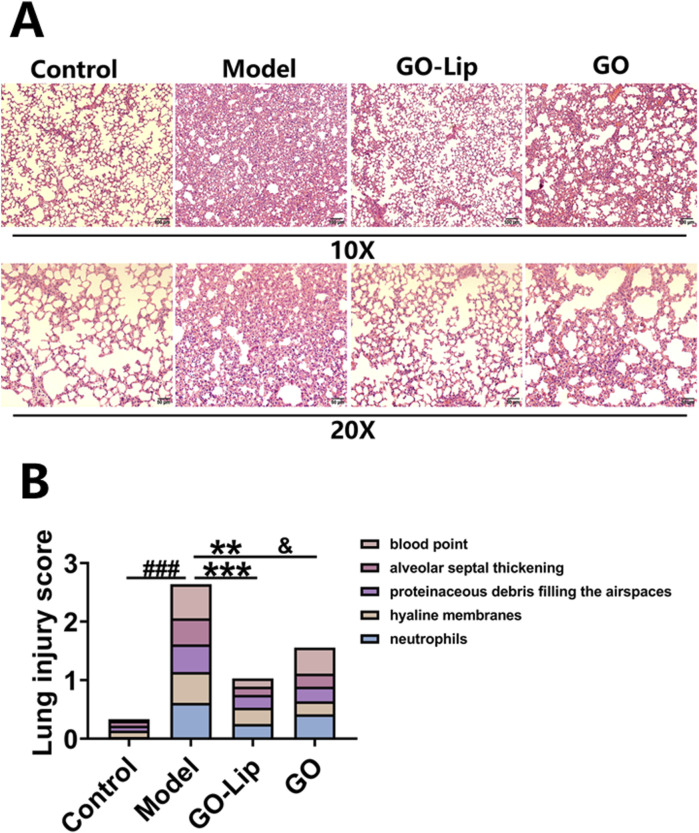
GO-lip effectively ameliorated LPS-induced ALI. **(A)** Representative H&E-stained lung tissue sections (magnification ×10, ×20 and scale bar 100 μm, 50 μm). **(B)** Pathology scores of lung tissues. The results confirmed that GO-lip can more effectively alleviate lung pathological lesions and reduce the lung injury score.

GO-lips significantly alleviated LPS-induced ALI in mice. Histopathological examination of lung tissue via H&E staining revealed that the mice in the control group exhibited a relatively intact alveolar architecture with minimal hemorrhage following intraperitoneal LPS administration, as shown in Figure 3. In contrast, the mice in the model group exhibited severe pulmonary damage characterized by extensive inflammatory cell infiltration within the alveolar spaces, marked congestion of lung tissue, and evident alveolar hemorrhage under microscopic observation. Administration of GO-lips, particularly in the liposomal formulation, attenuated these pathological changes, as evidenced by the reduced interstitial thickening, diminished neutrophil infiltration, and decreased number of hemorrhagic and congestive lesions. Notably, compared with the nonliposomal treatments, the GO liposomal formulation exerted a more pronounced anti-inflammatory effect against LPS-induced lung injury.

### GO-lips mitigated pulmonary edema in murine models

**FIGURE 4:**
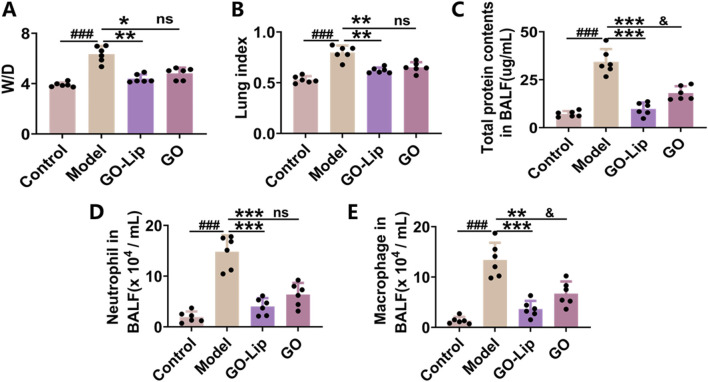
GO-lip mitigated pulmonary edema in murine models. The impact of GO-lip on lung tissue W/D **(A)**, lung index **(B)**, total protein concentration in BALF **(C)**, the number of neutrophil granulocytes **(D)** and macrophage **(E)** were in BALF was evaluated in LPS-induced ALI mice. Data are presented as means ± SDs. Statistical significance is indicated as follows: ^###^ *p* < 0.001 versus the control group; *p < 0.05, **p < 0.01, and ***p < 0.001 versus the model group; ^&^ *p* < 0.05 compared to the GO-lip group, n.s.: no significant. The results indicated that GO-lip can more effectively attenuate pulmonary edema and diminish the infiltration of inflammatory cells.

In mice, LPS stimulation induces pulmonary edema, which can be quantitatively assessed by calculating the lung wet-to-dry weight ratio and the lung coefficient. As shown in Figures 4A,B, the lung W/D in the LPS-treated model group was markedly greater than that in the control group (^###^ *p* < 0.001). However, the administration of GO-lips resulted in significant decreases in both the lung wet-to-dry weight ratio and the lung coefficient (**p < 0.01). Furthermore, LPS exposure increased capillary membrane permeability compared to that observed in the normal control group (^###^ *p* < 0.001), leading to the extravasation of macromolecular substances and a consequent significant increase in the protein concentration in BALF. Notably, the administration of GO-lips effectively decreased capillary membrane permeability, thereby substantially limiting the increase in the protein concentration in BALF (Figure 4C; ***p < 0.001), as well as neutrophil (Figure 4D; ***p < 0.001) and macrophage (Figure 4E; ***p < 0.001) numbers in mice BALF. Remarkably, GO encapsulated in liposomes exhibited an even greater capacity to reduce the protein content in alveolar lavage fluid.

### GO-lip mitigated the LPS-induced release of multiple cytokines in mice

**FIGURE 5:**
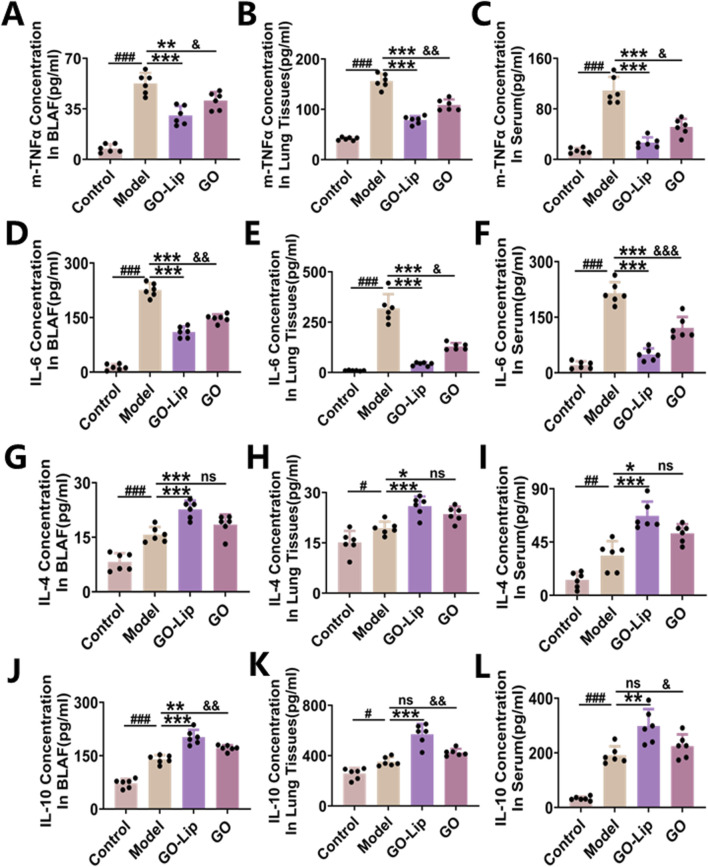
GO-lip mitigated the LPS-induced release of multiple cytokines in mice. In the context of LPS-induced acute lung injury, TNF-α **(A–C)**, IL-6 **(D–F)**, IL-4 **(G–I)**, and IL-10 **(J–L)** were measured. Data are presented as means ± SDs. Statistical significance is indicated as follows: ^###^ *p* < 0.001 relative to the control group; *p < 0.05, **p < 0.01, and ***p < 0.001 relative to the model group; and ^&^ *p* < 0.05, ^&&^ *p* < 0.01 relative to the GO-lip group, n.s.: no significant. The results confirmed that GO-lip can more effectively inhibit the secretion of pro-inflammatory cytokines and enhance that of anti-inflammatory cytokines.

ALI is characterized primarily by damage to lung tissue resulting from uncontrolled and progressively amplified inflammatory responses within the body. The activation and subsequent release of proinflammatory cytokines in lung tissue are crucial factors in the pathogenesis and progression of ALI and serve as key biomarkers for the systemic inflammatory status. In the present study, ELISAs were used to quantify the concentrations of proinflammatory cytokines, specifically TNF-α and IL-6, in both the BALF, lung tissue and serum of mice with LPS-induced ALI (Figures 5A–F). The findings demonstrated that LPS administration significantly increased TNF-α and IL-6 production compared to that in the blank control group (^###^ *p* < 0.001). Conversely, the administration of GO-lips markedly decreased the concentrations of these inflammatory mediators in mice stimulated with LPS (***p < 0.001). Additionally, the concentrations of IL-4 and IL-10, recognized as pivotal anti-inflammatory cytokines that can modulate excessive inflammatory responses and are produced by various cell types, including B cells and macrophages, were evaluated. Quantitative analysis via ELISA revealed modest increases in IL-4 and IL-10 secretion in the ALI model group (Figures 5G–L, ^#^ *p* < 0.05, ^###^ *p* < 0.001). Notably, GO-lip treatment significantly increased the secretion of these anti-inflammatory cytokines compared to that in the model group (^***^ *p* < 0.001). Furthermore, at equivalent doses, GO encapsulated in liposomes had a more pronounced inhibitory effect on LPS-induced pulmonary inflammation and the production of inflammatory mediators than did unencapsulated GO.

### GO-lips attenuate oxidative stress induced by LPS in murine models

**FIGURE 6:**
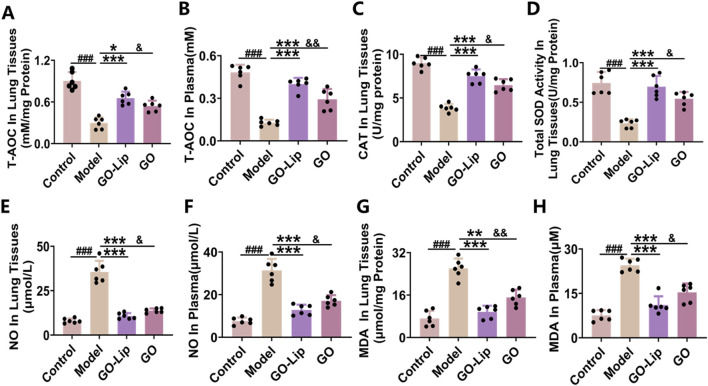
GO-lip attenuate oxidative stress induced by LPS in murine models. In the context of LPS-induced ALI, the levels of T-AOC **(A,B)**, CAT **(C)**, SOD **(D)**, NO **(E,F)**, MDA **(G,H)** were measured. Data are presented as means ± SDs. Statistical significance is indicated as follows: ^###^ *p* < 0.001 versus the control group; *p < 0.05, **p < 0.01, ***p < 0.001 versus the model group; ^&^ *p* < 0.05, ^&&^ *p* < 0.01 versus the GO-lip group, n.s.: no significant. The results confirmed that GO-lip more effectively alleviate oxidative stress-induced damage and restore antioxidant capacity.

A comprehensive review of the relevant literature indicated that oxidative stress interacts with inflammatory responses, leading to cellular damage. Accordingly, in the present study, the protective effects of GO-lips against LPS-induced oxidative stress were initially assessed by measuring the T-AOC and the activity of SOD and CAT in both lung tissue and serum from mice. Compared with the control and treatment groups, the LPS model group exhibited significant decreases in the T-AOC (Figures 6A,B, ^###^ *p* < 0.001) and in the expression levels of the antioxidant enzymes CAT (Figure 6C, ^###^ *p* < 0.001) and SOD (Figure 6D, ^###^ *p* < 0.001). Notably, the administration of 50 mg/kg GO-lips markedly increased antioxidant enzyme activity and restored the total antioxidant capacity (***p < 0.001). Building upon these results, we further investigated the effect of GO-lips on oxidative stress in mice with LPS-induced ALI. The concentrations of the oxidative stress markers MDA and NO in lung tissue and serum were quantified using MDA and NO assays. The LPS-induced ALI group presented significantly increased concentrations of NO (Figures 6E,F, ^###^ *p* < 0.001) in the plasma and lung interstitium, as along with increased concentrations of MDA (Figures 6G,H, ^###^ *p* < 0.001), a lipid peroxidation end product, indicating pronounced oxidative stress. Treatment with GO-lips effectively mitigated LPS-induced oxidative stress by significantly reducing the MDA and NO levels (***p < 0.001). Importantly, at an equivalent dose, the therapeutic effect of GO encapsulated in liposomes was superior to that of unencapsulated GO.

### GO-lips activate the Nrf2 signaling pathway in mice with ALI

**FIGURE 7:**
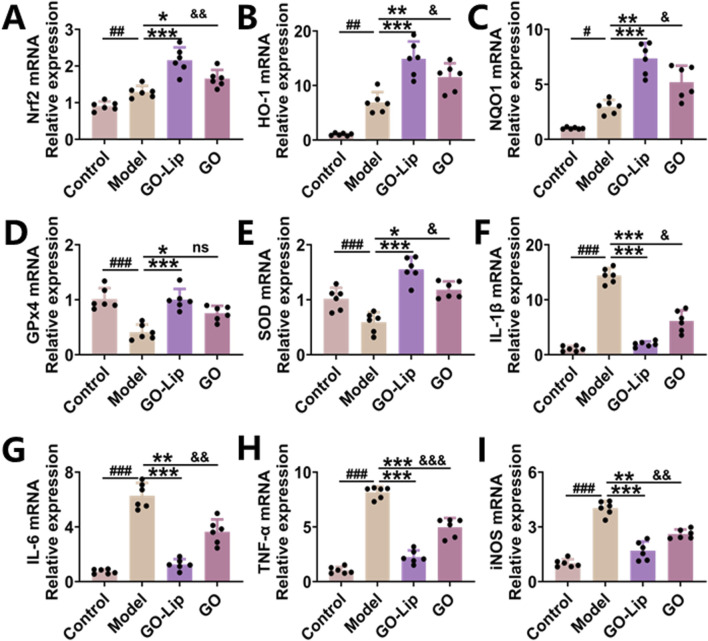
GO-lip inhibited oxidative stress and inflammatory responses via the Nrf2 signaling pathway. **(A–I)** Nrf2, HO-1, NQO1, GPx4, SOD, IL-1β, IL-6, TNF-α, iNOS, mRNA were detected using qRT-PCR. Data are presented as means ± SDs. Statistical significance is indicated as follows: ^###^ *p* < 0.001 versus the control group; *p < 0.05, **p < 0.01, ***p < 0.001 versus the model group; ^&^ *p* < 0.05, ^&&^ *p* < 0.01, ^&&&^ *p* < 0.001 versus the GO-lip group, n.s.: no significant. The results confirmed that GO-lip more effectively activated Nrf2 signaling pathway to combat LPS-induced oxidative stress and inflammation responses.

The Keap1–Nrf2 signaling pathway is the main antioxidant defense mechanism against environmental stress-induced damage. To evaluate the anti-inflammatory and antioxidant mechanisms of GO-lips, RT‒qPCR was used to quantify the mRNA expression levels of key components of relevant signaling pathways, including Nrf2, HO-1, NQO1, GPX4, and SOD. The RT‒qPCR results revealed that following ALI induction, GO-lip treatment elicited more pronounced activation of the Nrf2 pathway than did free GO treatment, thereby increasing the antioxidant capacity *in vivo* (Figures 7A–E, ^&^ *p* < 0.05, ^&&^ *p* < 0.01). The generation of ROS induces the nuclear translocation of p65, which subsequently upregulates the expression of proinflammatory mediators such as TNF-α, IL-1β, IL-6, and iNOS. Notably, treatment with GO-lips significantly decreased the levels of these proinflammatory cytokines in murine models, consistent with our prior ELISA results (Figures 7F–I, ^&^ *p* < 0.05, ^&&^ *p* < 0.01, ^&&&^ *p* < 0.001). Collectively, these data suggest that the therapeutic effects of the GO-lip formulation on LPS-induced ALI may be attributed, at least partially, to its ability to activate the Nrf2 signaling pathway.

### Molecular dynamics simulation of Nrf2 and compounds in GO

**FIGURE 8:**
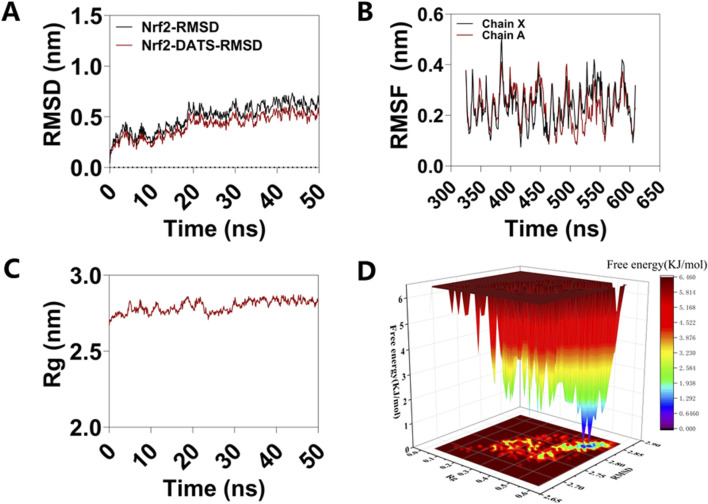
MD simulation of Nrf2-DATS in treatment of ALI. **(A)** RMSD curves of Nrf2-DATS complexes; **(B)** RMSF curve of Nrf2-DATS complexes; **(C)** Rg curves of Nrf2-DATS complexes; **(D)** 3D Gibbs free energy landscape of the Nrf2-DATS complex. MD simulations affirmed that DATS stably binds to Nrf2.

To comprehensively examine the anti-oxidative stress effects of GO, we elucidated the ligand-binding interaction between DATS, the principal small molecule component of GO, and the Nrf2 protein. Additionally, MD simulations were conducted to assess the stability and flexibility of the Nrf2–DATS complex. As shown in Figure 8A, the RMSD metric was employed to evaluate the structural stability of the complex throughout the MD simulations [18]. The all-atom MD simulations, performed over a duration of 50 ns, demonstrated that the Nrf2–DATS complex maintained its structural stability, with the backbone RMSD consistently remaining within an acceptable range and exhibiting minimal fluctuations during the simulation period. The RMSF was used to assess variations in the complex at the residue level [19]. The RMSF values indicated differential flexibility across residues within the Nrf2 binding pocket, with the majority of the residues involved in the interaction with DATS exhibiting reduced flexibility (<0.6 Å), suggesting increased rigidity upon ligand binding (Figure 8B).

Rg, which is correlated with protein volume and tertiary structure, serves as a critical parameter for evaluating protein stability in biological systems [20]. Elevated Rg values are indicative of increased ligand flexibility and diminished system stability, whereas lower Rg values correspond to a more compact and well-organized protein conformation. As shown in Figure 8C, the Rg value of Nrf2 remained stable throughout the MD simulation, with an average value of between 2.5 and 3.0 nm.

The Gibbs free energy landscape (FEL) provides insight into the stability of a receptor–ligand complex, where lower Gibbs free energy values denote greater complex stability [21]. By utilizing the RMSD and Rg values, a 3D Gibbs free energy landscape was constructed to identify and analyze the steady-state conformational characteristics of the complex. The regions depicted in blue and purple within the landscape correspond to stable conformational states exhibiting the minimal free energy. The 3D Gibbs free energy landscapes of the Nrf2–DATS complex are presented in Figure 8D. These visualizations reveal that the complex predominantly occupies a low Gibbs free energy state when the Rg value is between 0.5 and 0.6 nm and the RMSD value is between 2.75 and 2.85 Å.

## Discussion

ALI and ARDS represent sequential stages along the same pathological continuum. ALI constitutes the initial phase of the disease, which, upon further clinical deterioration, progresses to ARDS [22]. The recent COVID-19 pandemic has resulted in a significant increase in ALI incidence, underscoring the high mortality associated with this condition and the current lack of effective pharmacological interventions [23]. Currently, the most effective management approach for ALI is protective ventilation, and lung-protective ventilation combined with optimized supportive care is the preferred treatment modality. However, extended application of lung-protective ventilation may result in VILI, thereby worsening the patient’s clinical status. Consequently, the identification and development of novel therapeutic agents are critical for reducing ALI-related mortality and improving patient quality of life. GO, a commercially available extract derived from natural garlic, has been demonstrated to modulate multiple signaling pathways and to exert broad biological effects across various disease models. LPS, a principal pathogenic component of Gram-negative bacteria, is an important etiological factor in ALI. LPS exposure induces the excessive production of cytokines, chemokines, and reactive oxygen species, thereby precipitating extensive pulmonary damage through inflammatory cascades [24–26]. The LPS-induced ALI model is widely utilized in experimental research because of its ability to rapidly reproduce acute injury to pulmonary epithelial and endothelial barriers as well as the acute inflammatory response within the airways [27]. Preclinical models play an essential role in elucidating pathogenic mechanisms and assessing therapeutic interventions; however, their translational value depends largely on the extent to which they accurately reproduce human disease conditions. Intraperitoneal (i.p.) administration of LPS can be used to model sepsis-associated acute lung injury (SA-ALI). Upon intraperitoneal delivery, LPS gains access to the systemic circulation, triggering a cascade of innate immune responses that culminate in systemic inflammatory response syndrome (SIRS). This inflammatory state frequently progresses to multiple organ dysfunction syndrome (MODS), which is characterized by impaired function of the pulmonary, hepatic, renal, and cardiovascular systems. This pathological progression constitutes the primary clinical manifestation of ALI commonly encountered in ICU settings [28, 29]. This particular model was employed in the current study to facilitate the translation of findings from preclinical investigations to clinical applications within the ICU setting.

Liposomes, which are spherical vesicular carriers composed primarily of phospholipid bilayers and cholesterol, have attracted considerable attention as drug delivery systems because of their structural similarity to cellular membranes. This similarity endows them with excellent biocompatibility, facilitates the targeted accumulation of therapeutic agents at sites of pathology, minimizes adverse effects associated with off-target drug distribution, maintains the pharmacological activity of the encapsulated drug during metabolism and circulation, and increases drug bioavailability [30, 31]. In addition, the unique structure of liposomes allows it to more easily cross the physiological barrier of the lungs to accomplish drug delivery [32]. By regulating their particle size between 100 and 200 nm, they can reduce the rapid clearance of the endothelial system to maintain the required blood drug concentration throughout the body [33]. Liposomal delivered drugs has played an importent therapeutic role in different lung diseases by passively and actively targeting the lesion site, such as viral pneumonia [34], COPD [35], lung cancer [36, 37] and so on. For GO with poor solubility and low bioavailability, the solubility and bioavailability of the drug can be greatly improved by being encapsulated in the hydrophobic core of the liposome, which in turn enhances the therapeutic efficacy of the GO. GO loaded liposomes can directly enter the bloodstream through intravenous injection. Average particle size of GO loaded liposomes is 175 ± 3 nm, they can reduce the rapid clearance of the endothelial system to maintain the required blood drug concentration by passively targeting the lesion site in ALI.

This study demonstrated that following intraperitoneal administration of LPS, mice predominantly exhibited reduced feeding and drinking behaviors, lethargy, an inability of the forelimbs to support the body, piloerection, limb tremors, hypothermia, and incontinence. Conversely, treatment with GO-lips resulted in increased activity levels and increased food consumption in the mice. Histopathological examination of lung tissues via light microscopy revealed that the lung architecture in the control group mice remained normal and well defined, with no discernible pathological alterations. In contrast, the mice in the LPS-induced model group exhibited severe pulmonary injury and inflammation characterized by extensive infiltration of inflammatory cells within the lung interstitium, thickening of the alveolar septa, disruption of alveolar integrity, and evident intra-alveolar hemorrhage and congestion. Administration of GO-lips markedly attenuated LPS-induced pulmonary damage and inflammatory responses. During the early phase of ALI, substantial infiltration of inflammatory cells impairs alveolar epithelial cell function, resulting in the accumulation of proteins and fluids within the alveoli and lung interstitium instead of their clearance via lymphatic drainage, ultimately leading to pulmonary edema [38–40]. Accordingly, we assessed the extent of pulmonary edema and the integrity of the alveolar‒capillary barrier by quantifying the lung W/D, lung coefficient, BALF protein concentration, and neutrophil and macrophage numbers in BALF. The findings indicated that LPS-induced ALI significantly increased the W/D, lung coefficient, BALF protein concentration, and neutrophil and macrophage numbers in BALF compared with those in the control group. Treatment with GO-lips significantly reduced these values, thereby decreasing intra-alveolar accumulation of fluid. These effects are critically important for preserving the pulmonary microenvironment and improving the clinical outcome of ALI.

Acute fulminant inflammation is a pathological hallmark of ALI. Research has indicated that during the initiation, amplification, and persistence phases of ALI-associated inflammation, alveolar macrophages undergo polarization toward the M1 phenotype within the proinflammatory microenvironment. These M1 macrophages subsequently secrete proinflammatory cytokines such as TNF-α, interleukin-1β (IL-1β), and interleukin-6 (IL-6), which facilitate the mobilization, recruitment, and activation of neutrophils, monocytes, and other effector immune cells [41]. TNF-α serves as a critical early mediator of inflammation and is rapidly released at the onset of the inflammatory process. Additionally, TNF-α stimulates the release of other inflammatory cytokines, including IL-1β and IL-6, thereby amplifying the inflammatory cascade [42, 43]. IL-6, which is secreted predominantly by macrophages, is a key proinflammatory cytokine involved in various pulmonary inflammatory disorders [44]. Clinical observations in patients with ALI or sepsis revealed significantly increased concentrations of TNF-α and IL-6 in BALF, which correlated with progressive respiratory dysfunction [45]. Consistent with this finding, resident alveolar macrophages in murine models become polarized toward the M2 phenotype, and this transition is accompanied by the production of diverse anti-inflammatory cytokines that facilitate inflammation resolution and promote local tissue repair [46]. M2 macrophages contribute to the accumulation of specific anti-inflammatory cytokines, notably interleukin-10 (IL-10), which is recognized as a principal anti-inflammatory mediator capable of suppressing the production of multiple proinflammatory factors. Elevated IL-10 levels indicate an enhanced anti-inflammatory response, mitigating tissue damage induced by proinflammatory cytokines [47]. Interleukin-4 (IL-4) also functions as an important anti-inflammatory cytokine, broadly inhibiting the expression of various proinflammatory mediators, including TNF-α and IL-6, and has been demonstrated to promote IL-10 secretion [48]. In the present study, administration of GO-lips via tail vein injection resulted in significant reductions in the TNF-α and IL-6 concentrations compared with those in the model group, along with markedly increased IL-4 and IL-10 secretion. These findings substantiate the ability of GO-lips to attenuate both systemic and local pulmonary inflammatory responses in murine models of endotoxin-induced ALI.

Oxidative stress commonly accompanies inflammation and can exacerbate inflammatory responses, whereas inflammatory mediators can, in turn, further promote oxidative stress. Under physiological conditions, ROS levels are maintained in a dynamic equilibrium through balanced production and elimination. However, external stimuli can induce excessive ROS generation, disrupting the clearance of oxygen free radicals and leading to oxidative imbalance, thus contributing to various pathological states [49]. In the context of ALI/ARDS, the phenomenon known as the respiratory burst results in excessive ROS production, which suppresses endogenous antioxidant defenses and causes oxidative damage [50]. Numerous studies have identified SOD and catalase (CAT) as key antioxidant enzymes capable of scavenging superoxide anions within cells or organisms. Their activity levels serve as indirect indicators of the cellular antioxidant capacity. Conversely, the level of MDA, a lipid peroxidation product, reflects the accumulation of free radicals and the extent of lipid peroxidation [51, 52]. The overall antioxidant capacity of a biological system is determined by the combined levels of antioxidant macromolecules, small molecules, and enzymes, collectively measured as T-AOC, which is a crucial parameter for assessing oxidative stress [53]. During oxidative stress, NO functions as an important biological signaling molecule that can react with free radicals to form nitrite (NO2-), a reactive nitrogen species (RNS) with potent oxidizing properties [54]. In the present study, treatment with GO-lips resulted in elevated expression of SOD and CAT, an increased T-AOC, and reduced concentrations of MDA and NO, suggesting a balanced oxidative–antioxidative state in treated mice. Conversely, the mice in the LPS-induced model group exhibited the opposite profile, indicating pronounced oxidative stress. These findings demonstrate that GO-lips possess significant antioxidant capacity and effectively suppress ROS production.

Inflammation and oxidative stress are interrelated processes. Importantly, apoptosis is critically involved in the pathogenesis of ALI. During the initial phase of LPS-induced ALI, injured alveolar epithelial cells and activated macrophages release ATP via pannexin channels [55]. This extracellular ATP functions as a damage-associated molecular pattern (DAMP), thereby activating purinergic signaling pathways [56]. This activation not only stimulates the formation of the NLRP3 inflammasome, promoting the maturation and secretion of IL-1β, but also induces the production of ROS, exacerbating the reciprocal amplification between inflammatory responses and oxidative stress. Consequently, a cascade of inflammatory mediators activates apoptotic signaling pathways and disrupts mitochondrial homeostasis. Simultaneously, the reaction of ROS and NO generates peroxynitrite, leading to lipid peroxidation, protein and DNA damage, and further activation of apoptotic mechanisms [57].

The complex interplay among inflammation, oxidative stress, and apoptosis highlights the pivotal role of redox imbalance in the pathogenesis of ALI. This redox imbalance is precisely modulated via the Keap1–Nrf2 signaling pathway, which serves as the principal cellular defense mechanism against oxidative damage. The Keap1–Nrf2–ARE signaling pathway constitutes a fundamental mechanism in the regulation of cellular redox balance and the response to xenobiotic stress. Its principal role involves protecting cells against oxidative damage, environmental toxins, and deleterious chemical agents through the activation of transcriptional programs that upregulate cytoprotective genes. Under normal conditions, the transcription factor Nrf2 is retained in the cytoplasm through its interaction with Keap1 and is subjected to ubiquitin-mediated proteasomal degradation. Upon exposure to oxidative stress, Nrf2 escapes degradation and is translocated to the nucleus, where it binds to small Maf proteins and activates the transcription of genes containing antioxidant response elements (AREs) [58]. These processes results in the robust transcription of downstream antioxidant genes, including those encoding HO-1, NQO1, GPX4, and SOD. In summary, Nrf2 mitigates oxidative stress-induced damage by promoting the transcription of antioxidant genes, thereby performing a vital protective function in the pathogenesis of ALI. Additionally, the Nrf2/HO-1 signaling pathway mediates anti-inflammatory effects through the suppression of proinflammatory mediators such as NLRP3, TNF-α, IL-1β, and IL-6 [59, 60]. Consistent with the findings of previous studies, our findings revealed elevated mRNA expression levels of Nrf2, HO-1, and NQO1 after ALI induction. The administration of garlic oil-loaded liposomes markedly increased the expression of Nrf2, HO-1, NQO1, GPX4, and SOD while concurrently reducing the mRNA levels of IL-1β, TNF-α, IL-6, and iNOS.

Molecular simulation is a robust methodology for elucidating the stability and dynamic behavior of protein‒ligand complexes. In this study, a 50 ns MD simulation was conducted to analyze the dynamic characteristics of the Nrf2–DATS complex. The MD simulation data, particularly the RMSD, RMSF, and Rg values, provided critical indicators for evaluating the stability of the Nrf2–DATS complex as well as the conformational integrity of the Nrf2 protein’s tertiary structure following its interaction with small molecules, including assessments of amino acid residue hydrophobicity. The MD simulation results confirmed that DATS binds stably to Nrf2, thereby facilitating the activation of the Nrf2/HO-1/NQO-1 signaling pathway.

However, further investigations are needed to determine whether GO-lips can directly target pulmonary epithelial cells and pulmonary endothelial cells to exert protective effects and improve delivery efficacy.

## Conclusion

**FIGURE 9:**
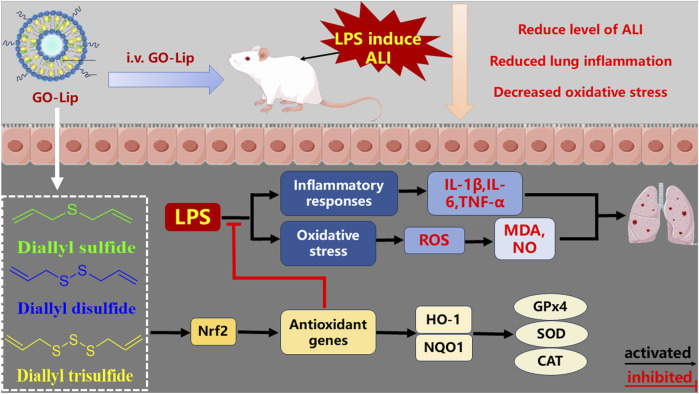
The schematic illustration of anti-ALI effects of GO-Lip. In ALI mice, LPS induced inflammation and oxidative stress, which damaged lung tissues. However, GO-Lip protects against LPS-induced ALI attenuates inflammation and oxidative stress by activated Nrf2 signaling pathway.

In summary, this study focused initially on the development of GO-lips as a novel nanodrug delivery system. *In vivo* experiments were subsequently performed to assess the therapeutic efficacy of this formulation against LPS-induced ALI in Figure 9. The findings indicated that GO-lips markedly increased the therapeutic efficacy of GO. In particular, GO-lips exhibited antioxidant properties mediated through the activation of the Nrf2 signaling pathway, which in turn upregulated the expression of downstream effectors such as HO-1 and NQO1. This mechanism contributed to the attenuation of inflammatory responses induced by oxidative stress and resulted in downregulated expression of proinflammatory genes, including TNF-α and IL-6. Finally, these molecular and cellular effects culminated in the amelioration of pathological alterations in lung tissue architecture.

## Data Availability

The raw data supporting the conclusions of this article will be made available by the authors, without undue reservation.
